# Apparent Motion from Outside the Visual Field, Retinotopic Cortices May Register Extra-Retinal Positions

**DOI:** 10.1371/journal.pone.0047386

**Published:** 2012-10-15

**Authors:** Martin Szinte, Patrick Cavanagh

**Affiliations:** 1 Laboratoire Psychologie de la Perception, Université Paris Descartes, Sorbonne Paris Cité, Paris, France; 2 Centre National de la Recherche Scientifique, UMR 8158, Paris, France; Ecole Polytechnique Federale de Lausanne, Switzerland

## Abstract

Observers made a saccade between two fixation markers while a probe was flashed sequentially at two locations on a side screen. The first probe was presented in the far periphery just within the observer's visual field. This target was extinguished and the observers made a large saccade away from the probe, which would have left it far outside the visual field if it had still been present. The second probe was then presented, displaced from the first in the same direction as the eye movement and by about the same distance as the saccade step. Because both eyes and probes shifted by similar amounts, there was little or no shift between the first and second probe positions on the retina. Nevertheless, subjects reported seeing motion corresponding to the spatial displacement not the retinal displacement. When the second probe was presented, the effective location of the first probe lay outside the visual field demonstrating that apparent motion can be seen from a location outside the visual field to a second location inside the visual field. Recent physiological results suggest that target locations are “remapped” on retinotopic representations to correct for the effects of eye movements. Our results suggest that the representations on which this remapping occurs include locations that fall beyond the limits of the retina.

## Introduction

With our head and eyes steady, our normal binocular vision covers a visual field of about 200 to 220 degrees of visual angle [1]. In order to extend that limited area and mostly to bring several objects of interest to our central vision we frequently move our eyes and heads (up to 5 times per second for the eyes, see [2]), abruptly shifting each time the projections on our retinas. Active cortical processes have been discovered in several visuo-motor areas (e.g., LIP, SC, FEF) that predict the retinal locations that attended objects will have following each eye movement [3]–[7]. This visual areas are organized in retinotopic coordinates [8], [9], so this updating process, called “remapping” [3], keeps track of target locations in the world despite the constant shifts on the retina. These processes may take advantage of a copy of the motor command for each eye movement (efference copy or corrolary discharge, [10]–[12]) to predict the new, post-saccadic target location.

In this paper, we ask what happens to the representation of a target when remapping specifies a location outside the visual field, as would happen for any target near the edge of our visual field when we make a saccade away from that target. Will the target still have an active representation despite the extra-retinal location? We can easily imagine that we maintain target representations in memory for objects we have seen but that are no longer in view [13]–[15]. But we are not addressing whether visual memory encodes locations in general space around the body including behind the head, we are examining whether active perception itself does so. To test this, we will use a motion paradigm where observers report whether or not a probe appears to move even though one position falls, after an eye movement, outside the visual field.

We adapt a simple apparent motion task that we developed to assess the accuracy of the compensation (remapping) for saccades [16]. In the original study, we presented two dots, one before and one after a horizontal saccade. Because of the eye movement, the two dots are separated by a large horizontal shift on the retina in addition to a vertical shift due to their actual displacement in the world. Despite this oblique displacement on the retina, participants reported seeing motion being close to vertical, almost as it is on the display monitor, demonstrating an efficient compensation for eye movements.

In our study here, we simply move the first dot to the edge of the visual field and follow it by a saccade away from its location. In order to update that location on a retinotopic representation, it must be remapped outside the visual field, because, if it were still present after the saccade, its location in space would now fall beyond the limit of the retina.

We reported here that observers do see apparent motion across a saccade even though its first location falls outside the visual field by the time the second position is presented. Since the first and second presentations are chosen to fall at approximately the same retinal location (probe displacement is matched to saccade amplitude), the perception of motion suggests that apparent motion is computed in spatial not retinal coordinates. This result has also been reported in several previous articles [16]–[18] and it indicates that the compensation for the saccade must occur prior to the inference of motion. If this compensation or remapping [19], [20] occurs even when it would transfer activity to a location effectively outside the visual field, it could be evidence for the existence of *visual* cells that represent extra-retinal space.

## Methods and Apparatus

### Observers

Ten volunteers from Université Paris Descartes took part in the experiment (all observers were naïve to the purpose of the experiment, age 20–29 years, 4 males, and 6 females). All had normal or corrected-to normal vision and gave informed consent. The experiment was carried out according to ethical standards specified in the Declaration of Helsinki and was approved by the Ethics Committee from the Université Paris Descartes. All observers gave written informed consent before participating in the experiment.

### Apparatus, Instruments and Stimuli

**Figure 1:**
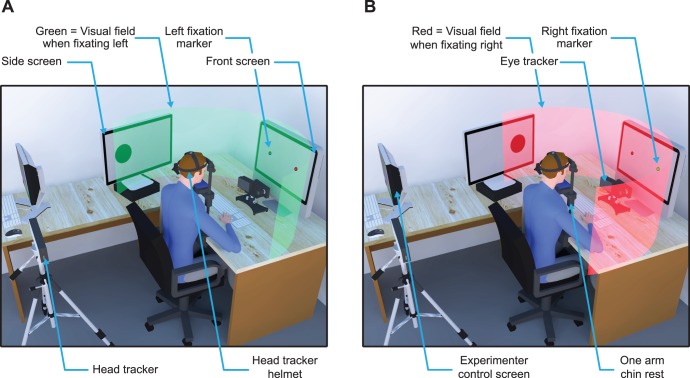
Apparatus and visual field. Two screens positioned at a distance of 60 cm from observers’ head were used, a front screen displaying the fixation (green dot) and saccade (red dot) markers and a side screen displaying the apparent motion probes. Eye and head position are monitored using an eye tracker combined with a head tracker and a chin rest with the left support removed in order to leave the side screen visible. (a) The green parabolic field represents observer’s visual field when fixating on the left fixation marker. The side screen is positioned such that the leftmost apparent motion probe falls just within the observer’s visual field when he or she is fixating the left marker on the front screen. (b) The red parabolic field represents observer’s visual field when he or she fixates the right fixation marker on the front screen. When fixating the rightmost marker, the right motion probe on the side screen falls at approximately the same position on the observer’s retina as the left probe does when fixating the left marker, even though the two probes do not have the same position in space. Note, however, that the position of left motion probe falls outside observer’s visual field when he or she fixates the right marker (at which time the left probe is no longer present).

Observers were seated in a quiet, dimly lit room. Fixation markers were 1°-diameter green (30.0 cd/m^2^) and red dots (30.0 cd/m^2^) on a gray background (100 cd/m^2^) presented on a gamma-linearized Apple iMac built-in 24″ TFT display (Cupertino, CA, USA) set 60 cm in front of observers’ eyes (see “front screen” in Figure 1a). Apparent motion probes were 4°-diameter black dots (0.1 cd/m^2^) on a gray background (100 cd/m^2^) presented on a gamma-linearized Apple 24″ LED-backlit TFT display (Cupertino, CA, USA) placed in observers’ left visual periphery (see “side screen” in Figure 1a) at a distance of 60 cm. Both screens had identical screen resolution and size (1920 by 1200 pixels covering 48.89° by 30.56° each), as well as identical refresh rate (60 Hz). The experiment was controlled by an Apple iMac Intel Core 2 Duo computer. Manual responses were recorded via a standard keyboard. The dominant eye’s gaze position was recorded and available online using an EyeLink 1000 Desktop Mounted (SR Research, Osgoode, Ontario, Canada) at a sampling rate of 1 kHz. Three-dimensional head orientations as well as three-dimensional head spatial locations were recorded using a LaserBird optical motion tracker (Ascension Technology Corporation, Burlington, VT, USA), at a refresh rate of 60 Hz. This head tracker is composed of a lightweight sensor worn on a helmet held fixed to the back of the head and of a fan-shaped laser beam scanner positioned 60 cm below observer’s head (see “head tracker helmet” and “head tracker” in Figure 1). The experimental software controlling the display, the response collection as well as the online eye and head tracking was implemented in Matlab (MathWorks, Natick, MA, USA), using the Psychophysics [21], [22] and EyeLink [23] and in-house head tracking toolboxes. Saccades were detected online when the gaze passed outside and landed later within virtual circles with a radius of 15% of the saccade amplitude (giving 3°; 3.75° or 4.5°-radius for 20°, 25° or 30° saccade trials) centered on the fixation and the saccade markers. Eye movement data were also re-analyzed offline based on eye velocity computed from subsequent samples in the eye position series [24]. The thresholds for peak velocity and minimum duration used for saccade detection were 3.0****SD and 20 ms. Head movement were detected online and trials were stopped and replayed later if observer’s head orientation changed of ±2.5 degrees of rotation (for either the yaw, roll or pitch angle) or if head position changed of ±2.5 cm in any direction from an initial head calibration angles and locations.

### Eye-head and Secondary Screen Calibration

At the beginning of each experimental block, observers were asked to turn their heads in the central axis of the front screen. The head tracker provided the initial three-dimensional head orientation and spatial position values that were then used to detect any later head movement, as well as to positioned observers’ head after any break. To help observers to maintain steady fixation, we used a chin rest that had only the right vertical support for the forehead brace so that the left support did not block the view of the side screen. Observers then executed a 13-point eye-tracking calibration in order to determine their gaze directions on the front screen. Then, we positioned the side screen such that its horizontal center fell 60 cm from observers’ head and such that half of the side screen was located outside observers’ left visual field when they correctly fixate at either 10°, 12.5° or 15° on the right side of the front screen center (see Figure 1b). To determine observer’s left visual field limit, we used a contrast detection task in which they had to report the contrast polarity, light or dark, of a 4°-diameter circle that could either be dark gray (25 cd/m^2^) or light gray (175 cd/m^2^) on a mid-gray background (100 cd/m^2^). The probe was positioned 2° to the left of the side screen center and based on observers’ discrimination, we moved the screen in order to increase or decrease the probe eccentricity. We stopped this procedure when observers said that they could not see anything on the side screen and when they were close to chance level on 20 consecutive trials (50% ± 5% correct). This adjustment procedure assured that observers were unable to see the left half of the side screen when they correctly fixated the right marker on the front screen (Figure 1b), although they can perfectly discriminate the probe contrast of that location on the left half of the side screen when fixating the left marker on the front screen (Figure 1a).

### Experimental Procedure

After the initial head and second screen calibration, all observers ran sequentially in a random order the 3 experimental conditions where the saccade amplitudes as well as the motion amplitudes vary between 20, 25 and 30°. Depending on the experimental condition, two fixation markers, one green and one red were presented at 10, 12.5 or 15° to the left and to the right of the front screen center, such that observers made horizontal saccades of either 20, 25 or 30°, selected equiprobably across trials. Observers were instructed to always fixate the green fixation marker and follow it as accurately as possible as it exchanged locations with the red one. The green fixation marker could appear at the beginning of a trial either on the left or the right of the front screen center, leading to equiprobable number of rightward and leftward saccade trials. Each trial began with the fixation marker filled with a smaller dark grey bull’s-eye. When the observer’s gaze was detected within a virtual circle centered on the fixation marker and if the head had not moved since the initial calibration, the bull’s-eye changed from grey to orange. The orange dot indicated that correct fixation was achieved and that the trial would start momentarily. After 600 ms of correct fixation the marker was entirely filled with green and the trial began. Each trial was composed of three back and forth cycles where the red and green markers exchanged position every 700 ms. In the two first cycles nothing was presented on the second screen positioned to the left side of observers’ eyes (side screen). This initial sequence helped observers to synchronize their saccades with the exchange of the two dots and to prepare themselves for the main cycle. In the main cycle, two apparent motion probes were presented sequentially on the side screen, one before and one after the saccade. Each probe was presented during 400 ms, with the first turned off 150 ms before the exchange of the green and red markers while the second turned on 150 ms later, giving then 300 ms for observers to complete their saccades. Probes were presented on the horizontal midline of the side screen and separated by the same offset as that between the fixation and saccade markers on that trial (20°, 25° or 30°). Finally, the order of appearance of these probes was equiprobably right-first or left-first, producing equal numbers of leftward and rightward motion trials. At the end of the main sequence, a red ring appeared around the green fixation marker indicating that the observers should report whether they saw or not any motion on the side screen.

**Figure 2:**
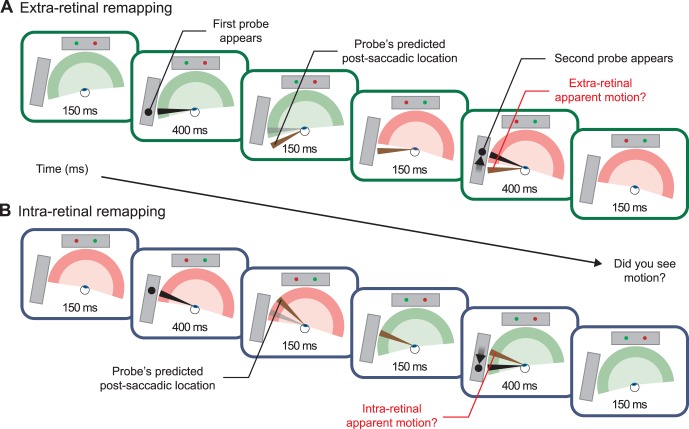
Stimulus sequence of the “intra-retinal” and “extra-retinal remapping” conditions. Each panel shows the sequence of the stimuli with a view from above the observer (represented by a single eye). The front screen and the side screen are shown as grey rectangles. The visual fields are shown as a green or red half circle when the observer fixates respectively the left or right fixation marker on the front screen. Each panel also represents the projections on the retina of the currently presented probe (black lines), of previously presented probe (gray lines) and of the predicted post-saccadic position of a probe (remapping) following the saccade (brown lines). (a) In the sequence of the extra-retinal remapping condition, probes were sequentially presented on the side screen, at the edge of observers’ visual field, 150 ms before and 150 ms after the fixation markers exchange locations. Observers were instructed to follow the green fixation marker, making rightward saccades while the peripheral probe move in the same direction. Observers report after each trial whether they saw motion on the side screen. Probes distance matched the fixation markers distance, such that the two probes fell closely at the same position on the retina even if they had two distinct positions in space. Under these conditions, to perceive motion between probes, the first probe should be remapped at its post-saccadic position on the retina, falling then outside observer’s visual field (see brown line), on extra-retinal visual space. (b) In the sequence of the intra-retinal remapping, observers made leftward saccades while probes moved in the same direction. The first probe is again remapped at its post-saccadic position, falling now inside observer’s visual field, on intra-retinal visual space.

**Figure 3:**
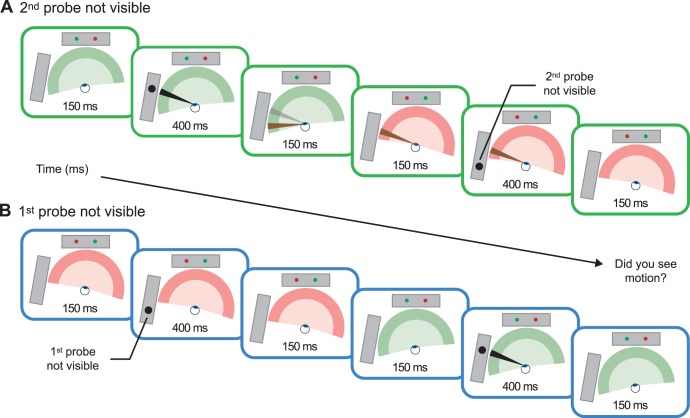
Stimulus sequence of the “2^nd^ probe not visible” and “1^st^ probe not visible” conditions. All conventions are the same as in Figure 2. These two control conditions tested whether we had properly positioned the side screen such that the left part of it lay outside observer’s visual field when he or she fixated the right fixation marker. (a) In the sequence of the “2^nd^ probe not visible” condition, observers made rightward saccade while probes on the side screen moved in the opposite direction (leftward). Although the first probe location fell inside observers’ visual field and may have been remapped (brown line), the second probe fell outside observer’s visual field, such that no motion could be perceived after the saccade. (b) In the sequence of the “1^st^ probe not visible” condition, observers made leftward saccade while probes moved rightward. Then, the first probe appeared outside of observer’s visual field such that no motion could be perceived after the saccade.

Because the side screen was positioned in such a way that its left half could not be seen when the observers fixated at the right fixation marker of the front screen, the combination of the two saccade directions and the two motion directions give the four experimental conditions described in Figures 2 and 3.

In the first condition (Figure 2a), both the saccade and probes are displaced rightward. In order to predict the spatial position of the first motion probe following the saccade, its position is remapped by the amplitude of the saccade, but in the opposite direction (see Movie 1: http://cavlab.net/ExtraretinalMovies). The spatial location of the first probe should therefore be remapped outside of observer’s visual field, if it can be. We called that condition, “*extra-retinal remapping*”. Note in this case, that on the retina, both the first and second probe fall at approximately the same location at the edge of observer’s visual field, one before and one after the saccade. The remapping is required to compute the retinal location where the first probe would have fallen after the saccade if it were still there, in order to detect any changes in its position that occurred at the same time as the saccade.

In the second condition (Figure 2b), observers executed a leftward saccade while motion probes moved also leftward on the side screen (see Movie 2: http://cavlab.net/ExtraretinalMovies). Again to predict the spatial position of the first motion probe following the saccade, its position is remapped by the amplitude of the saccade but in the opposite direction. In this case the remapped location of the first probe now falls inside the observer’s visual field. We therefore called this condition “*intra-retinal remapping*”.

In the third condition (Figure 3a), called “*2^nd^ probe not visible*” condition, the saccade goes rightward while probes moved leftward on the side screen (see Movie 3: http://cavlab.net/ExtraretinalMovies). In this case, although the first probe location may have been remapped, the second probe falls outside observer’s visual field, and thus no motion should be perceived after the saccade, testing whether we had properly positioned the side screen and thus the position of the probes. Finally, in the fourth condition (Figure 3b), called “*1^st^ probe not visible*”, observers executed a leftward saccades but this time the motion probes moved rightward on the side screen (see Movie 4: http://cavlab.net/ExtraretinalMovies). In this case, the first motion probe appeared outside of the observer’s visual field and thus no motion should be perceived after the saccade (again testing whether we had properly positioned the side screen).

## Results

We evaluated the proportion of motion reports for the 4 experimental conditions (described above) for all saccade amplitudes tested. When inaccurate saccade trials or trials where head movement exceeded our criteria from the initial head calibration position were detected on line, they were rejected and replaced (305 trials repeated out of 2705 trials played, giving 2400 selected trials). We then re-analyzed the eye-tracking data offline in order to select only trials where the motion probes were presented trans-saccadically. We thus looked for trials in which the saccade started after the first probe offset and ended before the second probe onset. Within addition we also rejected trials with blinks and those that failed a finer offline evaluation of saccade accuracy. This correction led us to reject a further 548 trials, leaving 77.2% of selected trials (1852/2400; corresponding to 68.5% of all trials). Note that maintaining a steady head position was not a major source of trial rejections as 84.7% of the rejections were due to saccades occurring too early (476 of 562 rejected trials). The screening left an average of 185±16 trials per observer, ranging from 45.4% (GL: 109/240) to 97.9% (JT: 235/240).

**Figure 4:**
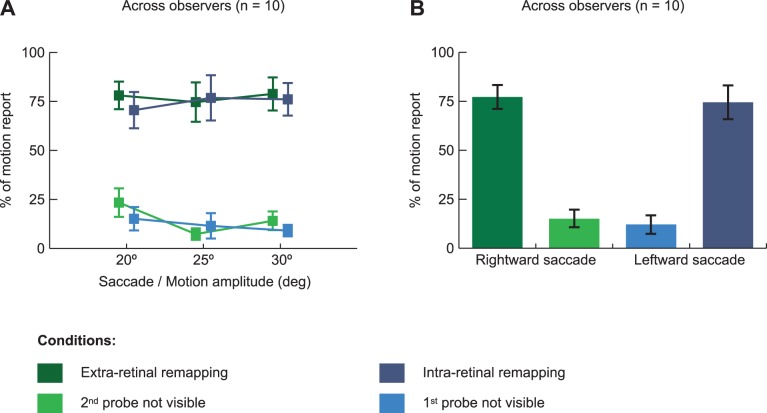
Group results. (a) The left panel shows the average (n = 10) percentage of motion report for the 4 experimental conditions and the three saccade and motion amplitudes tested. (b) The right panel shows the same data but this time collapsed across the three saccades and motion amplitudes tested. Error bars in both panels represent SEM across observers.

Figure 4a shows the proportion of motion reports for the four experimental conditions and the three different saccade/motion amplitudes. A repeated measures ANOVA (with experimental conditions and saccade amplitudes as main factors), shows a main effect of experimental condition (F(3,27) = 40.74, *p*<0.001) and no effect of the saccade amplitude (F(2,54) = 0.67, *p* = 0.52). There is no significant interaction between these variables (F(6,54) = 0.92, *p* = 0.49). We therefore collapsed our data across the different saccade amplitudes (Figure 4b). In both “extra-retinal” and “intra-retinal remapping” conditions observers report seeing motion on the side screen in 3 trials out of 4, with 77.3% ± 6.1% and 74.5% ± 8.5% of motion report across all observers for “extra-retinal” and “intra-retinal remapping” tests, respectively (no significant difference between the two conditions, F(1,9) = 0.39, *p* = 0.55). These results are significantly different from the control conditions (F(1,9) = 47.11, *p*<0.001), where motion was reported in about 1 or 2 trials out of 10, 15.0% ± 4.6% for the “2^nd^ probe not visible” condition and 12.0% ± 4.8% for the “1^st^ probe not visible” condition (no significant difference between the two control conditions, F(1,9) = 1.22, *p* = 0.30). In these two control conditions, one or the other of the two probes should not have been visible so we would expect no report of motion. These reports may arise from response errors and guessing but are most likely a consequence of the very liberal criterion we gave to observers. They were instructed to report any kind of motion they saw in the far periphery on the side screen. Post-experimental debriefing revealed that some observers reported both seeing motion and seeing one probe on some trials. They explained that they considered a small jitter of the single probe seen in far periphery as being motion to report. Nevertheless, some of these motion reports in the control conditions could reflect errors in our calibration of the visual field limit. This would suggest that for at most 15% of the trials, the more peripheral stimulus still fell within the visual field. Even this maximum possible level of miscalibration cannot account for the motion reports on 77% of the extra-retinal remapping trials when the location of the first probe, originally within the visual field, is supposed to fall outside the visual field following the eye movement.

**Figure 5:**
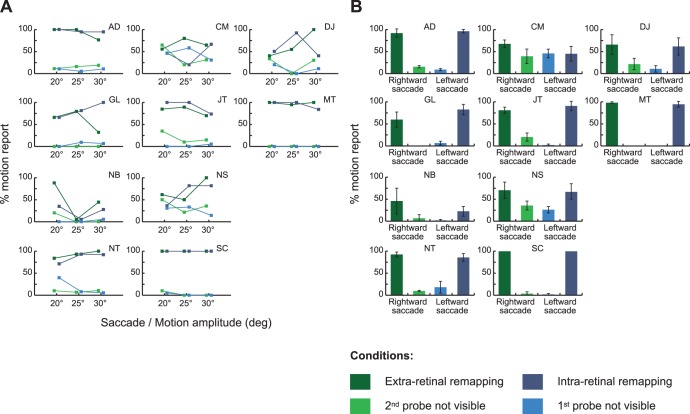
Individual results. This figure shows (a) for each observer (in columns and rows), the percentage of motion report for the four experimental conditions and the 3 saccade and motion amplitudes tested and (b) these same data collapsed across saccade amplitudes. Error bars in panel (b) represent SEM across the 3 saccade and motion amplitude tested.

Next, we determined whether the pattern seen across observers also held in individual results. Figure 5 shows the proportion of motion reports across our four experimental conditions and saccade/motion amplitudes for each observer individually (see Figure 5a), as well as the same data collapsed across saccade amplitudes (see Figure 5b). Individual data are similar to group results across 8 of the 10 observers: the motion reports when both probes were visible (extra and intra-retinal remapping) were significantly more frequent than in the two control conditions where only one probe was visible (all *p*<0.05, one-tail t-tests). However, 2 observers, CM and NB, showed a different pattern of results. CM showed similar frequencies of motion reports in the conditions with two visible dots vs one (t(1) = 1.71, *p* = 0.16). At the end of the experiment, CM explained that she experienced motion even when she saw only a single dot. In that case she perceived motion as briefer and of shorter amplitude than when she saw two dots, but reported it as motion nonetheless. On the other hand, NB rarely reported seeing motion in any condition (t(1) = 3.27, *p* = 0.05). At the end of the experiment, we asked her to report if she saw apparent motion when the probes were displaced while she maintained fixation at the left marker (so that both probes were visible and moved without any intervening saccade). Interestingly, she didn’t report seeing motion suggesting that the distance between probes might have been too large or the delay between the two probes to long for her to see motion.

**Figure 6:**
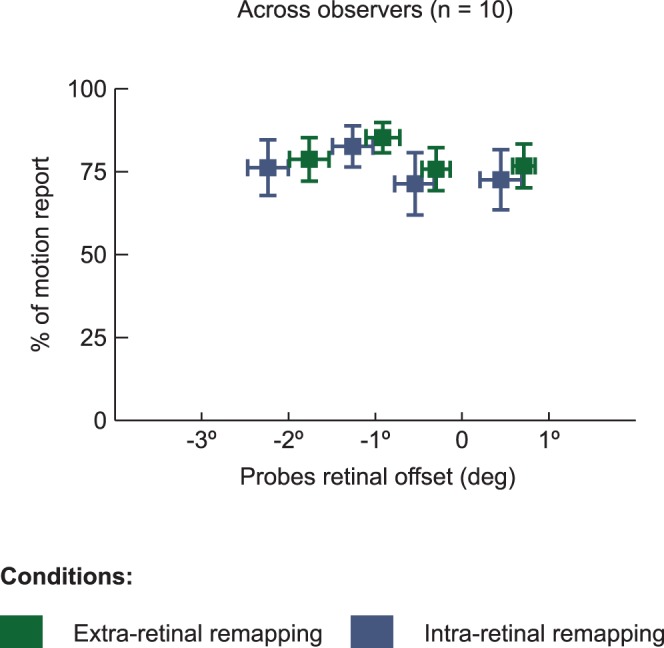
Effect of retinal offset between the two probes. This graph shows the proportion of motion reports for the retinal offset between the two probes (in quartile bins) for 2 experimental conditions where two probes were visible, averaged across observers (n = 10). With the head steady and the intended amplitude of the saccade (20, 25 or 30°) matching the amplitude between probes on the side screen, the two probes should fall on the same location on the observers’ retina. There is, however, a retinal offset that results from under- or overshoot of the saccade. As seen here, saccades generally landed short of the fixation target, leading to a retinal offset between –2 and +1 degrees. However, this retinal offset had no effect on observers’ motion reports. This is especially interesting for offsets around 0° when both probes stimulate almost the same retinal location (third quartile). Here observers report seeing motion across the saccade in both the extra and intra retinal remapping condition despite the lack of shift on the retina. Horizontal error bars represent SEM across observers for the retinal offset, while the vertical bars represent SEM across observers for the motion report.

Finally, we were interested to test if apparent motion could be seen when the same location on the retina was stimulated by the first and second probe when a saccade intervened. This duplicated Rock & Ebenholtz’s [18] demonstration of motion reports when a stimulus landed on the same retinal location before and after a saccade, although in our case, these locations are now in the far periphery. There, as here, observers reported seeing motion (see Figure 6) indicating that the location of the pre-saccadic probe was corrected for the saccade so that a large offset in space was seen rather than the null offset on the retina. Figure 6 shows the proportion of motion report for 4 quartiles bins of probes retinal offset in function of the 2 experimental conditions where two probes were visible. For the two experimental conditions separately, the proportion of motion report is very similar between the four different quartiles (ANOVA with motion shift quartiles and experimental condition as main factors shows no main effect of motion shift, F(3,63) = 1.01, *p* = 0.40). Critically, in both the extra and intra-retinal remapping conditions, when the probes’ retinal offset was around 0° (third quartile in Figure 6, retinal offset between −0.28° ± 0.16° and −0.58° ± 0.24° respectively), observers still reported seeing motion with, respectively, 75.8% ± 6.5% and 71.4% ± 0.2% of motion report across all observers. This last result shows that even when both probes stimulate approximately the same retinal location, observers report seeing motion across saccades. This indicates, as Rock and Ebenholtz [18] first reported, that apparent motion is seen in spatial coordinates, not retinal coordinates.

## Discussion

We used sequential probes with an intervening saccade to determine whether apparent motion was seen in spatial not retinal coordinates at the edge of the visual field. Critically, in one condition, the first probe fell within the visual field only when it was first presented, but its location then fell outside the visual field after the saccade. Nevertheless, on 75% of trials, motion was seen after the saccade from this extra-retinal location to a new location within the visual field, indicating that the visual system keeps track of locations that move outside the visual field due to saccades. Motion was also seen at the same rate (75% of trials) when the first probe location still fell within the visual field after the saccade.

Apparent motion was reported in these conditions even though the size of the saccades and the displacement of the probes were matched to produce, ideally, no displacement on the retina. In fact, of course, saccade may under- or overshoot but when we analyzed the frequency of motion reports, it was constant, independent of the actual small offset on the retina (from −2° to +1°) caused by inaccurate saccade landings, including the small range around no displacement. This result replicates the finding of Rock & Ebenholtz [18] demonstrating that apparent motion is determined in spatial not retinal coordinates.

In the remaining two conditions, either the first or second probe fell outside the visual field when it was presented and should have been invisible. The frequency of motion reports here was much lower (12 to 15%) indicating that our original calibration to locate the edge of the visual field was accurate.

Our results show that objects that move in the world are seen to move even if there is no displacement on the retina [18]. This indicates that the pre-saccadic location of the first probe is corrected for the effects of the saccade prior to the determination of apparent motion between the two locations. This correction removes the most of the effect of the saccade from the perceived motion direction (but not all, see [16]). The possible mechanisms for this compensation include “remapping” based on efference copy [19], [20], [11], [25], [12]. In our displays, when a probe is near the limit of the visual field and the saccade moves away from the probe, its predicted post-saccadic location is remapped outside the visual field, requiring extra-retinal representation. Apparent motion is then seen from this predicted post-saccadic location to the new probe location, back within the visual field. To explain our results, the representation would have to extend at least 15 degrees of visual angle outside the visual field. The representation of the far periphery (from 80 to 100°) covers very little cortical surface [26] so the extra 15° would take up even less. Indeed, in an fMRI study by Tark & Curtis [27] a persistent neural activity have been shown in FEF for memorized auditory stimuli presented in extra-retinal visual space, that is to location where no saccade could have been made.

Alternate proposals for saccadic correction when applied to our probes in the far periphery would also lead to a requirement for extra-retinal representation. For example, with “reference object theory,” a memory of the saccade target landscape is used to locate the original saccade goal so that no efference copy is needed to predict its location [28]–[31]. Since this process only involves the saccade target, other targets like our motion probes would have to be localized relative to the saccade target. After the saccade is made, the relative offset from the saccade target to the first probe then specifies a location outside the visual field. When the second probe appears at a new location, apparent motion is seen from the first position (outside the visual field) to the second.

In our procedure, we used apparent motion, a type of visual motion that for large displacements cannot be explained by simple motion receptors [32] and that is best described as an attentional phenomenon [33], [34]. As Wertheimer [34] described it, a probe first attracts attention to one location followed by a second probe that drags attention to its new location, giving a strong impression of motion. In the case of trans-saccadic apparent motion, attention would be first remapped to the expected post-saccadic location of the first probe, then when the second probe appears at a different location, attention is dragged to that new location even though the two probes were matched in retinal coordinates. This creates apparent motion in a spatiotopic reference frame [16], [17], supporting Rock and Ebenholtz’s [18] earlier report.

The perceptual effect we report differs from the more general ability to remember the location of an object previously seen but no longer visible. In our case the perception of motion suggests that basic visual representations of location underlie the effect rather than memory of location. Even without intervening saccades and possible extra-retinal locations, apparent motion is not seen for probe-to-probe intervals beyond about 400 ms [35] indicating that visual memory alone cannot produce apparent motion phenomena.

Our results suggest that positions outside the visual field are coded in saccade and attention maps, however, we cannot determine in this experiment whether the effective extra-retinal location actually corresponds to the spatial location of the first probe. Alternatively, all remapping or predicted locations that would lie beyond the edge of the visual field may simply be referred to the edge of the visual field. In this case, our stimuli would give an impression of a motion path half as long as the actual path. In a follow up experiment, we plan to ask observers to report the length of the motion path, and point to the first dot location to determine if there is any compression of locations at the edge of the visual field. Alternatively, we could add a vertical displacement to one of the two probes and ask observers to report the direction of the motion from which we could geometrically extract the length (as suggested by our reviewer).

### Conclusion

We show here that the perception of motion is reported between two probes when a saccade intervened between the presentation of the first and second probe, even though, in some conditions, when the second probe was presented, the effective location of the first probe lay outside the visual field. This result suggests that apparent motion can be seen from a location outside the visual field to a second location inside the visual field. The probe locations were arranged so that the shift between two distinct positions in space caused them to fall at approximately same position on the retina. The fact that apparent motion was seen under these conditions indicates that the motion is seen in spatial not retinal coordinates [18] and that therefore, the pre-saccadic probe location must be corrected for the effect of the saccade before the computation of the motion. This correction or “remapping” [3], [19], [20] would place the expected post-saccadic location of the first probe outside the visual field.

Our interpretation rests on the subjective motion reports of our observers. In both conditions where the first and second probes are presented within the visual field, observers report motion on about 75% of the trials and this figure is unaffected by whether the first probe’s location lies outside the visual field after the eye movement. So we believe that observers are reporting the phenomenal experience of motion, as requested, and not just reporting the displacement of the perceived (and remembered) locations. Displacement reports (not based on motion) would have reached 100% in these conditions with both probes visible. We do not believe that the eye movement itself is triggering a percept of motion as the great majority of cases with one of the two probes not visible led to reports of no motion.

Nevertheless, it is clear that this is only a first evaluation of extra-retinal motion percepts and that further studies that go beyond these subject motion reports are needed. If our first results here hold up, it suggests that areas representing visual stimuli in retinotopic coordinates have cells that respond to extra-retinal space, beyond the margins of the visual field. These cells keep track of targets that have just moved outside the visual field. If these stimuli then move to return to our field of view, we see them not as simply reappearing but as moving.
